# Efficacy and Tolerability of Stable Hybrid Cooperative Complexes of High- and Low-Molecular Weight Hyaluronic Acid in Asian Patients: An Open-Label Study

**DOI:** 10.1007/s00266-025-05188-x

**Published:** 2025-09-08

**Authors:** Suzanne Wei Na Cheng, Shi Yu Derek Lim, Clara Cigni, Franco Grimolizzi, Chee Leok Goh

**Affiliations:** 1 National Skin Centre, 1 Mandalay Road, Singapore, 308205 Singapore; 2 IBSA Farmaceutici Italia, Lodi, Italy

**Keywords:** Asian, Skin regeneration, Face, Hybrid cooperative complexes, Hyaluronic acid, Profhilo^®^

## Abstract

**Background:**

Few studies of Profhilo^®^ for facial skin regeneration in Asian patients exist.

**Aims:**

The efficacy and safety of Profhilo^®^ in regenerating facial skin in Asian females were investigated in a single-center study.

**Patients/Methods:**

Patients were treated with Profhilo^®^ on Weeks 0 and 4 and followed up on Week 12. Assessments included three-dimensional photography, Facial Volume Loss Scale (FVLS), Wrinkle Severity Rating Scale (WSRS), superficial skin hydration, transepidermal water loss (TEWL), skin elasticity, and self-rated product tolerability.

**Results:**

Of 30 enrolled Chinese females (aged 38.0–60.0 years), outcome data were analyzed for 28 and 26 patients at Weeks 0–4 and follow-up, respectively, aside from skin hydration (*N *= 8 and *N *= 6, respectively). Treatment led to a FVLS (*P *= 0.0150), WSRS (*P *= 0.0057), and *R*3 (“tiring effect” of the skin; *P* = 0.0424) decrease over time, but pairwise time point comparisons for these measures were not significant (NS). A trend toward a decrease in *F*1 (elasticity) over time occurred but was NS. A statistically significant improvement in superficial skin hydration and TEWL occurred over time (*P* = 0.0010 and *P* = 0.0055, respectively) and, specifically, at Week 12 versus baseline (*P* = 0.0034 and *P* = 0.0029, respectively). Patients reported “good” product tolerability.

**Conclusions:**

Profhilo^®^ for facial skin regeneration was efficacious and tolerable in Chinese patients. This study contributes to the limited available clinical data with Profhilo^®^ and Asian facial skin regeneration. Profhilo^®^ significantly improved superficial skin hydration and transepidermal water loss in Chinese patients. Profhilo^®^ was well tolerated in Chinese patients.

**Level of Evidence IV:**

This journal requires that authors assign a level of evidence to each article. For a full description of these Evidence-Based Medicine ratings, please refer to the Table of Contents or the online Instructions to Authors www.springer.com/00266.

## Introduction

Skin aging results in the progressive atrophy of the dermis and changes in the architectural organization of the skin, leading to folds and wrinkles, and as the most visible organ, the skin can show the first signs of aging [1, 2]. Skin aging is a multifactorial process caused by internal mechanisms (e.g., chronological age, genetics, hormonal changes, and inflammation) and external factors (e.g., ultraviolet [UV] exposure and pollution) leading to structural and physiological skin changes [1, 3, 4]. Clinical signs of skin aging include wrinkles, sunspots, uneven skin tone, and skin laxity [1]. Differences in skin aging by ethnicity exist [1, 5, 6]. These include genetic and molecular skin tissue differences, and muscle and bone anthropometric ethnicity-related variations [7]. People with Asian or African skin are reported to have a thicker and more compact dermis than those with Caucasian skin, and this thickness is proportional to the degree of pigmentation [1]. Although the Asian population is diverse, some studies have reported that Asians have a weaker facial skeletal framework than other ethnic groups, leading to greater gravitational soft tissue descent of the mid-face, malar fat pad ptosis, and tear trough formation [1]. Additionally, Asian versus Caucasian skin was reported to have similar basal transepidermal water loss (TEWL) and ceramide levels, but upon mechanical challenge, Asian skin was shown to have the weakest barrier function [6]. In a line-field confocal optical coherence tomography imaging study comparing facial skin aging in 109 Asian females in China (aged 20–70 years) with 100 Caucasian females of the same age range in France, distinct biological features specific to each population were found [5]. Asian females had statistically significant higher dermal–epidermal junction undulation, greater nuclei compactness, and lower cell network atypia versus similar aged Caucasians across all age groups [5]. The Asian cohort also had a statistically significantly thicker stratum corneum for all age ranges, and significantly thicker viable epidermis layer for most age groups in the cheekbone area of the face [5].

The main objectives of antiaging approaches are delaying the progress of skin senescence or mitigating the severity of age-related changes [2]. Aside from surgical approaches, strategies for managing skin aging include the use of UV protection, energy-based devices, topical agents, and injectables (e.g., injections of hyaluronic acid [HA], poly-L-lactic acid, polycaprolactone, polynucleotide, or botulinum toxin) [4]. The use of injectables for skin aging has increased to improve rhytides and restore soft tissue volume [4]. Currently, HA is the most used injectable for skin aging due to its biocompatibility, ease of use, and reversibility [4]. The use of HA injections is also rising; from 2022 to 2023, there was a 29.1% increase in the use of these injections in cosmetic procedures globally [8]. However, due to rapid degradation by hyaluronidase, native HA in the skin has a half-life of 24–48 h [9, 10]. To prolong dermal stability, chemically stabilized cross-linked HA fillers are available, which have been developed using cross-linking agents, such as 1, 4-butanediol diglycidyl ether [9]. Although 1, 4-butanediol diglycidyl ether is the safest and the most utilized cross-linking agent for this purpose, modern filler technologies are designed to lower its concentration and, hence, increase injectable biocompatibility [9].

Profhilo^®^ (IBSA Farmaceutici Italia Srl) is a medical device designed for intradermal use based on stable, hybrid cooperative complexes (HCCs) of high- and low-molecular weight HA produced with NAHYCO^®^ Hybrid Technology (an innovative thermal process that does not involve chemical reagents) [9, 11, 12]. This product consists of 32 mg of high-molecular weight HA (1100–1400 kDa) and 32 mg of low-molecular weight HA (80–100 kDa) in an injectable concentration (64 mg in 2 mL) [9]. In addition to its high concentration, this injectable shows ideal manageability, optimal tissue diffusion, low viscosity, and a tan delta > 1 (for greater fluidity versus elasticity) [12]. Three years of post-marketing experience with Profhilo^®^ on > 40,000 patients demonstrated that this product was safe and well tolerated [12]. Several published studies^1–6^ demonstrated the efficacy and safety of Profhilo^®^ for facial skin regeneration [7, 9, 13–16]. Nonetheless, aside from one comparative analysis, only one study (based in Italy) included Asian (Chinese) patients [7, 15]. Investigating cosmetic injectables in diverse skin types is important, as patients with skin of color represent a growing market for cosmetic injectables [17]. Furthermore, these patients can have different esthetic goals and treatment responses [17].

An open-labeled, exploratory, single-center study was performed to assess the efficacy and safety of HCCs of H-HA and L-HA (Profhilo^®^) for facial skin regeneration in Asian females and its ability to improve facial volume, wrinkle severity, skin hydration, and elasticity.

## Materials and Methods

### Eligibility Criteria

Eligible study participants were healthy females of Asian ethnicity (Chinese, Malay, Indian, or other Asian) who were between 38 and 60 years of age and willing to maintain their current diet, exercise regime, makeup, cosmetics, and facial wash routine, as well as good sun protection habits throughout the study. Participants included in the study were also willing to attend patient visits without makeup. Patients were also excluded from the study if they were pregnant or breastfeeding, had dermatological diseases affecting the face, had esthetic facial treatments in ≤ 12 months before study enrollment, participated in a similar trial in the previous 3 months, had permanent facial fillers, had a known sensitivity to Profhilo^®^ or its ingredients, or had significant active comorbidities. Participants who were receiving anti-inflammatory drugs, antihistamines, topical/systemic corticosteroids, narcotics, antidepressants, or immunosuppressive drugs in the preceding 4 weeks before the start of the study, as well as those with a medical/surgical record of using a medication that may interfere with the study, were not permitted to participate in the trial.

### Study Design and the Injection Procedure

**Fig. 1:**
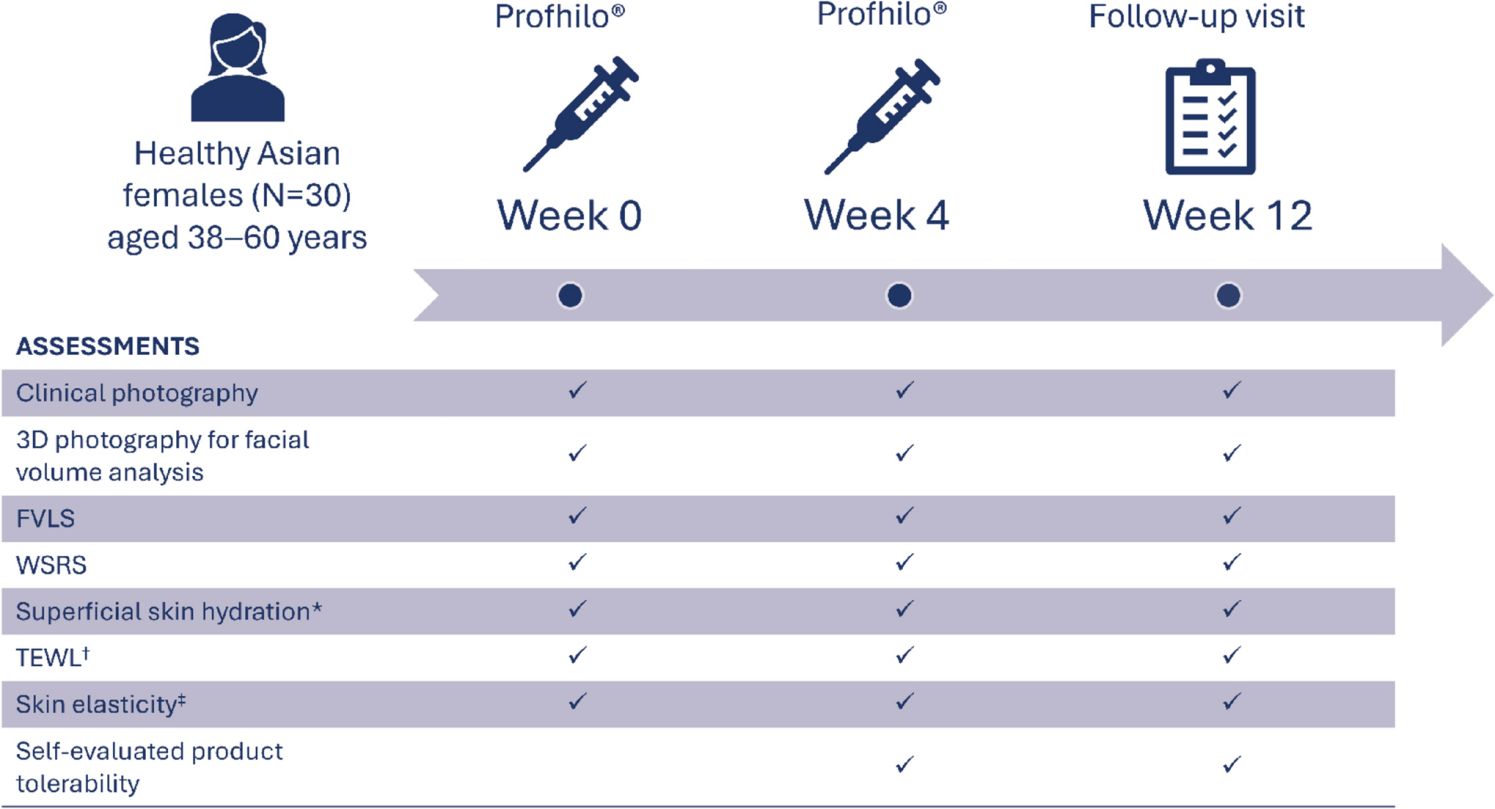
Study design. ^*^Measured using a Corneometer^®^ CM825. ^†^Assessed by a Tewameter^®^ or a VapoMeter^®^. ^‡^Evaluated using a Cutometer^®^. *3D* three-dimensional; *AEs* adverse events, *FVLS* Facial Volume Loss Scale; *TEWL* transepidermal water loss; *WSRS* Wrinkle Severity Rating Scale

Patients (enrolled in the study between November 2022 and February 2024) were treated with HCCs of H-HA and L-HA (Profhilo^®^) in two treatment sessions, 4 weeks apart (Fig. 1).

The product was administered intradermally into malar/submalar areas of the face using the Bio Aesthetic Point (BAP) technique per its instructions for use (IFU) [11]. This technique identifies five injection points on malar/submalar areas of both sides of the face (for a total of 10 points) [11], including the:

1. Zygomatic protuberance (≥ 2 cm away from the external side of the eye).
2. Nose wing (intersection point between a line drawn from the nose wing to the tragus and a perpendicular line from the pupil midline).
3. Tragus (1 cm in front of the lower margin of the tragus).
4. Chin region (1.5 cm toward the labial commissure from the intersection point between a vertical line in the middle of the chin and a perpendicular line drawn at a third of that line).
5. 1 cm above the mandibular angle.

HCCs of H-HA and L-HA (0.2 mL) were injected into each point on both sides of the face (at the dermis/subcutaneous level) using the bolus technique for the total administration of 2 mL of product [11]. A further follow-up visit was also conducted on Week 12.

### Assessments

Primary efficacy endpoints included facial volume, assessed by 3D photography for facial volume analysis (3D Camera with an analysis module, Vectra H1, Canfield, USA) and the Facial Volume Loss Scale (FVLS), and wrinkle severity, evaluated using the Wrinkle Severity Rating Scale (WSRS) [18, 19]. Secondary efficacy endpoints included superficial skin hydration evaluated by a Corneometer^®^ CM825 (Courage–Khazaka, Köln, Germany), transepidermal water loss (TEWL) measured using a Tewameter^®^ (Courage–Khazaka, Köln, Germany) and skin elasticity assessed using a Cutometer^®^ (Courage–Khazaka, Köln, Germany). Assessments of skin hydration and TEWL were performed for only eight patients, as the protocol was amended to add these assessments during the course of the study, when the authors anecdotally noted improvements in skin hydration in treated participants. These endpoints, as well as clinical photographs, were assessed at baseline (Week 0), Week 4, and Week 12. Patients also completed a questionnaire to assess their tolerability to the product on Weeks 4 and 12.

### Target Sample Size

According to a prior, similar study conducted in Italy [14] the mean difference in FVLS score between Week 0 and Week 16 was 0.6. Assuming a standard deviation of 0.6 and accounting for a possible dropout proportion of 10%, the sample size required to assess a change in FVLS score was estimated to be 19. The mean difference in WSRS score between Week 0 and Week 16 was 0.5 in the same study. Assuming a similar standard deviation of 0.6 and accounting for a possible dropout proportion of 10%, the sample size required to assess a change in WSRS score was estimated to be 27. Therefore, the target sample size for the study was 30 patients.

### Statistical Analysis

Absolute and relative frequencies were assessed for categorical variables, and means, standard deviations (SD), median values, and the interquartile range were calculated for continuous variables. The Shapiro–Wilk test was used to determine normality of distribution for outcome data obtained, with the repeated measures one-way ANOVA and Friedman tests subsequently used to assess statistical differences for normally and non-normally distributed data, respectively. Post hoc analyses were performed with the two-tailed paired *t*- and Wilcoxon matched-pairs signed rank tests for normally and non-normally distributed data, respectively.

A Holm–Bonferroni correction was applied for all post hoc analyses to account for multiple comparisons. Statistical analyses were performed using GraphPad Prism, version 10.2.1 (GraphPad Software, San Diego, CA, USA).

## Results

### Patient Demographics and the Number of Patients Included in the Analysis

Thirty females of Chinese ethnicity with a mean (standard deviation; SD) age of 49.3 (7.0) years and an age range of 38.0–60.0 years were included in the study (Table 1).

**Table 1:** Patient demographics and number of patients included in the analysis

Patient demographics	*N* = 30
Age (years)	
Mean (SD)	49.3 (7.0)
Range	38.0–60.0
Median (IQR)	50.0 (43.0–55.0)
Females, *n* (%)	30 (100.0)
Chinese ethnicity, *n* (%)	30 (100.0)
Enrolled patients, *n* (%)	30 (100.0)
Patients included in the final analysis for Weeks 0 and 4	28 (93.3)
Patients without 2 readings for each outcome measure at Weeks 0 and 4	2 (6.7)
Patients included in the final analysis for Week 12	26 (86.7)
Patients who breached the protocol^a^	3 (10.0)
Patients without facial volume images taken at Week 12	1 (3.3)

*IQR* interquartile range, *n* number of patients, *N* total number of patients, *SD* standard deviation.

^a^One participant had a chemical peel, another patient had double eyelid surgery, and it was later discovered that a third patient had laser surgery in the 12 months prior to the start of the study.

Two patients did not have two readings for each outcome measure on Weeks 0 and 4. Therefore, outcome data for 28 patients were analyzed during Weeks 0 and 4. In addition, three patients breached the study protocol and facial volume images were not taken for one patient during Week 12; therefore, outcome data for 26 participants were assessed during Week 12.

### Change in Facial Volume as Determined from Three-Dimensional Photograph Analysis

**Fig. 2:**
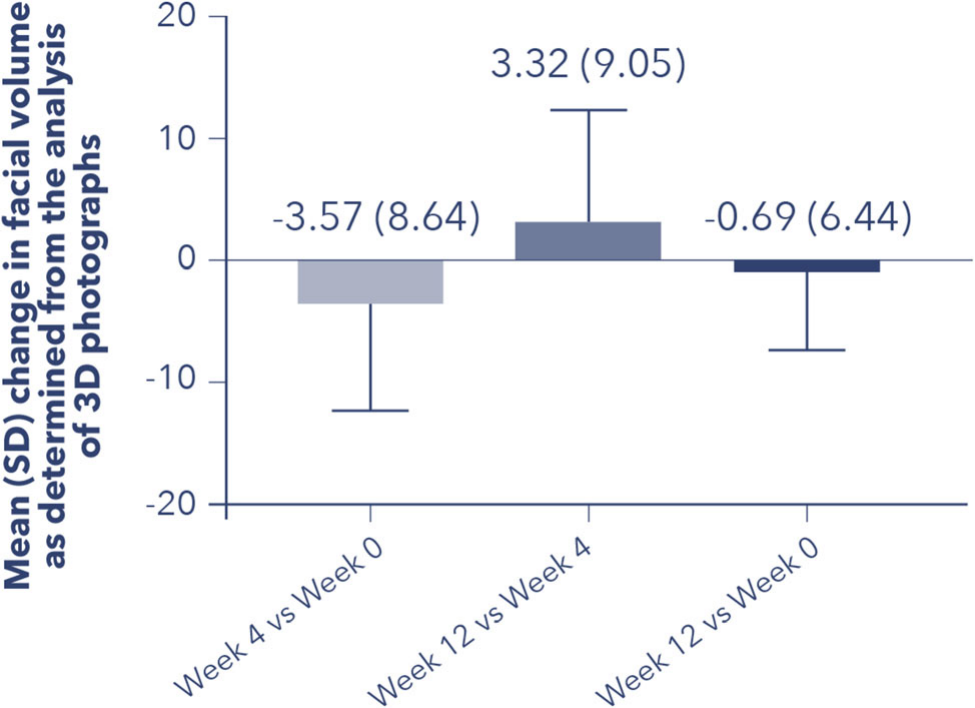
Change in facial volume as determined from the analysis of 3D photographs. *3D* three-dimensional; *SD* standard deviation. *N* = 28 for Week 4 versu Week 0, and *N* = 25 for Week 12 versus Week 4 and for Week 12 versus Week 0.

The mean change in facial volume, as assessed by the analysis of three-dimensional photographs, was more variable. Between Week 4 (post-treatment) and Week 0, there was a mean reduction in facial volume (mean [SD], − 3.57 [8.64]) as determined from the analysis of 3D photographs (Fig. 2).

However, this evaluation revealed an average increase in facial volume between Weeks 12 and 4 (mean [SD], 3.32 [9.05]) and a slight decrease in facial volume between Weeks 12 and 0 (mean [SD], − 0.69 [6.44]).

### FVLS and WSRS

Using the Friedman test, there was a statistically significant reduction in FVLS scores in 26 patients over time (*P* = 0.0150; Table 2), indicating at least one difference in medians among the time points.

**Table 2:** Facial Volume Loss Scale (FVLS) and Wrinkle Severity Rating Scale (WSRS)

	Wk 0 (*N* = 28)	Wk 4 (*N* = 28)	Wk 12 (*N* = 26)
FVLS			
Mean (SD)	2.71 (0.85)	2.64 (0.87)	2.46 (0.86)
Range	1.00–4.00	1.00–4.00	1.00–4.00
Median (IQR)	3.00 (2.00–3.00)	3.00 (2.00–3.00)	3.00 (2.00–3.00)

	*P*-value	*α*-level for *P*-value comparison^a^	Statistically significant?
Friedman test^b^	0.0150	0.0500	Yes
Wilcoxon matched-pairs signed rank test^c^			
Wk 4 versus Wk 0	0.5000	0.0500	No
Wk 12 versus Wk 4	0.1250	0.0250	No
Wk 12 versus Wk 0	0.0625	0.0167	No

	Wk 0 (*N* = 28)	Wk 4 (*N* = 28)	Wk 12 (*N* = 26)
WSRS			
Mean (SD)	2.82 (0.61)	2.75 (0.65)	2.54 (0.65)
Range	1.00–4.00	1.00–4.00	1.00–3.00
Median (IQR)	3.00 (3.00–3.00)	3.00 (3.00–3.00)	3.00 (2.00–3.00)

	*P*-value	*α*-level for *P*-value comparison^a^	Statistically significant?
Friedman test *P*-value^a^	0.0057	0.0500	Yes
Wilcoxon matched-pairs signed rank test *P*-value^b^			
Wk 4 versus Wk 0	0.5000	0.0500	No
Wk 12 versus Wk 4	0.0625	0.0250	No
Wk 12 versus Wk 0	0.0312	0.0167	No

*IQR* interquartile range, *FVLS* Facial Volume Loss Scale, *SD* standard deviation, *Wk* Week, *WSRS* Wrinkle Severity Rating Scale. ^a^Holm–Bonferroni correction was applied for post hoc analyses only (to account for multiple testing)

^b^Calculated using the Friedman test to assess whether there are significant differences in FLVS or WSRS scores among the three time points. The Friedman test could only be calculated for patients with no missing data points; therefore, 26 patients were included in the Friedman test

^c^Calculated using the Wilcoxon matched-pairs signed rank test and a two-tailed *p*-value. This statistical test only computes data that are available at both time points; therefore, for Wk 12 versus Wk 4 and Wk 12 versus Wk 0, 26 patients were included in the analysis.

However, post hoc analysis demonstrated that there was no statistically significant difference in median FVLS scores between Week 4 versus Week 0, Week 12 versus Week 4, or Week 12 versus Week 0.

For WSRS, the Friedman test showed a statistically significant reduction in scores in 26 patients over time (*P* = 0.0057; Table 2). Nonetheless, post hoc analysis showed no statistically significant difference in median WSRS scores between Week 0 versus Week 4, Week 12 versus Week 4, or Week 12 versus Week 0.

### Superficial Skin Hydration and TEWL

Superficial skin hydration (assessed by a Corneometer^®^) was evaluated only for eight patients at Weeks 0 and 4 and for six patients at Week 12. There was a statistically significant increase in superficial skin hydration values in 6 patients with complete data at all three time points over time (*P* = 0.0010; Table 3), as assessed using the repeated measures one-way ANOVA test.

**Table 3:** Superficial skin hydration (assessed by a Corneometer^®^) and TEWL (measured using a Tewameter^®^/VapoMeter^®^)

	Wk 0 (*N* = 8)	Wk 4 (*N* = 8)	Wk 12 (*N* = 6)
Superficial skin hydration			
Mean (SD)	77.58 (17.86)	82.58 (17.48)	93.71 (17.46)
Range	48.33–102.57	50.57–107.33	74.00–121.00
Median (IQR)	82.95 (60.23–88.70)	85.37 (71.21–94.31)	93.35 (76.18–108.00)
One-way ANOVA test *P*-value^a^	**0.0010**^**c**^		
Paired *t*-test *P*-value^b^			
Wk 4 versus Wk 0	**0.0153**^**c**^		
Wk 12 versus Wk 4	**0.0064**^**c**^		
Wk 12 versus Wk 0	**0.0034**^**c**^		

	Wk 0 (*N* = 28)	Wk 4 (*N* = 28)	Wk 12 (*N* = 26)
TEWL			
Mean (SD)	11.99 (3.90)	10.49 (2.95)	9.50 (2.02)
Range	6.87–25.93	3.13–15.50	6.40–13.33
Median (IQR)	11.33 (9.11–13.88)	10.42 (8.52–13.22)	9.44 (8.06–10.72)
One-way ANOVA test *P*-value^a^	**0.0055**^**c**^		
Paired *t*-test *P*-value^b^			
Wk 4 versus Wk 0	0.0572		
Wk 12 versus Wk 4	0.1217		
Wk 12 versus Wk 0	**0.0029**^**c**^		

*IQR* interquartile range, *SD* standard deviation, *TEWL* transepidermal water loss, *Wk* Week

^a^Calculated using the repeated measures one-way ANOVA test to assess whether there are significant differences in superficial skin hydration or TEWL values among the three time points. The repeated measures one-way ANOVA test could only be calculated for patients with no missing data points; therefore, 6 patients were included in the test calculation for superficial skin hydration and 26 patients were included in the test calculation for TEWL

^b^Calculated using the paired t-test and a two-tailed *p*-value. This statistical test only computes data that are available at both time points; therefore, for Wk 12 versus Wk 4 and Wk 12 versus Wk 0, 26 patients were included in the analysis. Bonferroni correction for multiple comparison was used (0.05/3)

^c^Significant values (< 0.05 for one-way ANOVA test and < 0.017 for the paired *t*-test rank test) are shown in bold font.

Post hoc analysis showed that there was a statistically significant 6% increase in median superficial skin hydration values for Week 4 versus Week 0 (*P* = 0.0153), a significant 13% increase in median superficial skin hydration values for Week 12 versus Week 4 (*P* = 0.0064), and a significant 21% increase in median superficial skin hydration values for Week 12 versus Week 0 (*P* = 0.0034).

For TEWL, there was a statistically significant difference in TEWL values in 26 patients over time (*P* = 0.0055; Table 3). Post hoc analysis showed that there was a statistically significant 21% reduction in median TEWL values between Week 12 versus Week 0 (*P* = 0.0029), but not for Week 4 versus Week 0 (*P* = 0.0572) or Week 12 versus Week 4 (*P* = 0.1217).

### Skin Elasticity

Skin elasticity parameters assessed using a Cutometer^®^ included *R*3 (a comparison between the maximum amplitude of the last and first curves, which indicates the skin’s “tiring effect”) and *F*1 (skin elasticity determined from the area of the curve) [20]. For *R*3, the lower its value is, the less fatigued the skin is [21]. For *F*1, the closer to 0 *F*1 is, the more elastic the skin is [20]. There was a statistically significant reduction in median *R*3 values in 26 patients over time (*P* = 0.0424; Table 4), assessed by the repeated measures one-way ANOVA test.

**Table 4:** Skin elasticity (assessed by a Cutometer^®^)

	Wk 0 (*N* = 28)	Wk 4 (*N* = 28)	Wk 12 (*N* = 26)
*R*3			
Mean (SD)	0.26 (0.10)	0.23 (0.08)	0.21 (0.05)
Range	0.11–0.50	0.11–0.42	0.12–0.35
Median (IQR)	0.24 (0.19–0.30)	0.23 (0.17–0.28)	0.21 (0.17–0.25)

	*P*-value	*α*-level for *P*-value comparison^a^	Statistically significant?
One-way ANOVA test^b^	0.0424	0.0500	Yes
Paired *t*-test^c^			
Wk 4 versus Wk 0	0.1160	0.0500	No
Wk 12 versus Wk 4	0.1156	0.0250	No
Wk 12 versus Wk 0	0.0238	0.0167	No

	Wk 0 (*N* = 28)	Wk 4 (*N* = 28)	Wk 12 (*N* = 26)
*F*1			
Mean (SD)	0.03 (0.03)	0.03 (0.03)	0.02 (0.02)
Range	− 0.02–0.08	− 0.01–0.16	− 0.02–0.08
Median (IQR)	0.03 (0.01–0.08)	0.03 (0.01–0.05)	0.02 (0.001–0.04)

	*P*-value	*α*-level for *P*-value comparison^a^	Statistically significant?
One-way ANOVA test^b^	0.0841	0.0500	No
Paired *t*-test^c^			
Wk 4 versus Wk 0	0.7782	0.0500	No
Wk 12 versus Wk 4	0.0816	0.0250	No
Wk 12 versus Wk 0	0.0177	0.0167	No

*F*1, Skin elasticity determined from the area of the curve (the closer to 0 *F*1 is, the more elastic the skin is); *IQR* interquartile range, *R*3, a comparison between the maximum amplitude of the last and first curves, which indicates the skin’s “tiring effect”; *SD* standard deviation, *Wk* Week

^a^Holm–Bonferroni correction was applied for post hoc analyses only (to account for multiple testing)

^b^Calculated using the repeated measures one-way ANOVA test to assess whether there are significant differences in *R*3 or *F*1 values among the three time points. The repeated measures one-way ANOVA test could only be calculated for patients with no missing data points; therefore, 26 patients were included in this test

^c^Calculated using the paired *t*-test and a two-tailed *p*-value. This statistical test only computes data that are available at both time points; therefore, for Wk 12 versus Wk 4 and Wk 12 versus Wk 0, 26 patients were included in the analysis.

However, post hoc analysis showed that there was no statistically significant difference in median *R*3 values between Week 4 versus Week 0 (*P* = 0.1160), Week 12 versus Week 4 (*P* = 0.1156), or Week 12 versus Week 0 (*P* = 0.0238).

For *F*1, there was no statistically significant difference in these values in 26 patients over time (*P* = 0.0841; Table 4). Additionally, post hoc analysis showed that there was no statistically significant difference in median *F*1 values between Week 4 versus Week 0 (*P* = 0.7782), Week 12 versus Week 4 (*P* = 0.0816), or Week 12 versus Week 0 (*P* = 0.0177).

### Product Tolerability as Rated by Patients

**Fig. 3:**
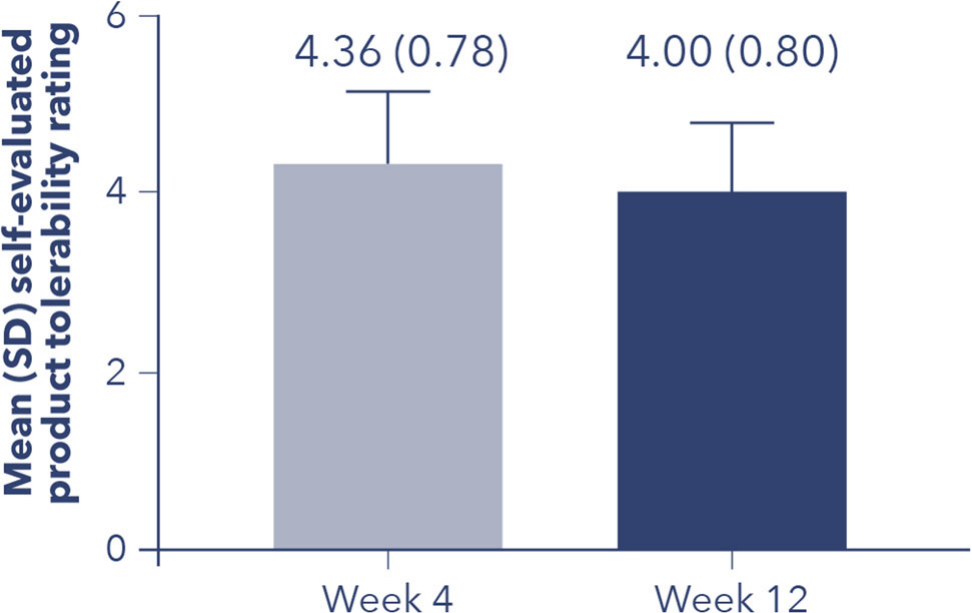
Product tolerability as rated by patients. Patients rated their tolerance to the product in a questionnaire which included the following options: (1) bad, (2) poor, (3) medium, (4) good, and (5) excellent

Patients rated their tolerance to the product in a questionnaire which included the following options: (1) bad, (2) poor, (3) medium, (4) good, and (5) excellent. At Week 4 and Week 12 (post-treatment), the mean (SD) self-evaluated product tolerability rating was 4.36 (0.78) and 4.00 (0.80), respectively, indicating that patients rated their tolerance to the product as good (Fig. 3).

## Discussion

Thirty Chinese females, aged 38.0–60.0 years, were included in the open-labeled, exploratory, single-center study to assess the efficacy and tolerability of two treatment sessions of HCCs of H-HA and L-HA (Profhilo^®^), administered four weeks apart, for facial skin regeneration. Efficacy data (aside from superficial skin hydration) were available for 28 patients on Weeks 0, 4 and 26 participants on Week 12. Superficial skin hydration was only available for eight patients on Weeks 0, 4 and six on Week 12. Product tolerability data were available for 28 patients on Week 4 and 26 participants on Week 12.

By three-dimensional photograph analysis, only a slight trend toward a decrease in mean (SD) facial volume of − 0.69 (6.44) from Week 0 to Week 12 was observed. Overall, although there was, as expected post-treatment with Profhilo^®^, a statistically significant decrease in FVLS (*P* = 0.015; Friedman test), WSRS (*P* = 0.0057; Friedman test), and the skin elasticity parameter, *R*3 (indicating the skin’s “tiring effect” [20]) over time (*P* = 0.0424; one-way ANOVA test), there was no statistically significant difference for these parameters by post hoc pairwise comparisons of time points, when a more stringent *p*-value to account for multiple testing (Holm–Bonferroni correction) was applied. Although a reduction in *F*1 (indicating degree of skin elasticity [20]) over time was expected following Profhilo^®^ treatment, the slight reduction observed on Week 12 versus baseline was not statistically significant. Treatment with Profhilo^®^ on this cohort of patients had the most substantial impact on skin hydration and TEWL, with a significant 21% increase in median superficial skin hydration (*P* = 0.0034; paired *t*-test) and a corresponding significant 21% reduction in median TEWL from baseline to Week 12 (*P* = 0.0029; paired *t*-test), despite the small number of patients with superficial skin hydration results (27% of enrolled patients had superficial skin hydration data on Weeks 0 and 4, and 20% of participants had those data on Week 12). On a scale of 1 (“bad”) to 5 (“excellent”), on average, patients rated their tolerability to the product as 4.36 on Week 4 and 4.00 on Week 12, indicating “good” product tolerability.

In a pilot study performed to assess the efficacy and tolerability of Profhilo^®^, administered using the BAP technique in two sessions (four weeks apart), in 28 Chinese women in Italy aged between 38 and 60 years, ten patients were treated in the face area and 18 participants received treatment in the neck area [15]. For patients in that study who were treated on their face, ten patients were assessed at baseline, Week 4, and Week 8, and three participants were evaluated at a Week 12 follow-up visit [15]. Although a statistical analysis was not performed for that study, Profhilo^®^ treatment in the face area resulted in a trend toward a 35% reduction in FVLS score at Week 4 and a 48% reduction at Week 8.^4^ Similarly, there was a trend toward a 22% reduction in WSRS score at Week 4 and a 29% reduction in this score at Week 8 [15]. Additionally, there was a trend toward a 2.6% increase in superficial skin hydration for the face area at Week 4 and a 11% increase in this parameter at Week 8 post-treatment [15]. No serious AEs were reported in that study and product tolerability was judged to be “good” or “excellent” by investigators [15]. In a comparative analysis conducted by Sparavigna et al. 2023, it was suggested that genetic and molecular skin tissue differences and muscle and bone anthropometric ethnicity-related variations might lead to more favorable baseline and efficacy outcomes following Profhilo^®^ treatment in Chinese versus Caucasian patients [7]. Although three separate studies [14, 15, 22] were considered in that analysis, two of which included evaluation of the face area, the authors found that baseline FVLS and WSRS scores were better in Chinese versus Caucasian patients [7]. It was also determined that FVLS and WSRS scores decreased by 48% and 29% in Chinese participants and by 21% and 12% in Caucasian patients, respectively, at Week 8 (after Profhilo^®^ treatment) relative to baseline [7]. Nonetheless, no statistical analysis was performed in that evaluation [7].

Studies in Asian patients with HA injections showed comparable efficacy for different fillers but lacked the benefits of HCCs combining H-HA and L-HA observed in our study, likely due to their key role in a regenerative approach [23–25]. HCCs of H-HA and L-HA can facilitate extracellular matrix homeostasis and maintain cellular viability to reverse signs of skin laxity via a bioremodeling action that works by improving the vitality of different cell types, including keratinocytes, fibroblasts, adipocytes, and myocytes [26]. Cellular and molecular changes in keratinocytes and fibroblasts in vitro and in a 3D skin model were compared following incubation of these cells with HCCs of H-HA and L-HA versus no treatment or H-HA or L-HA alone [26, 27]. HCCs of H-HA and L-HA in that study increased expression levels of collagen and elastin, suggesting a bioremodeling effect of the product on tissues, that most likely occurred because of the long-lasting release of H-HA and L-HA and their concurrent action [26, 27]. HCCs of H-HA and L-HA in that in vitro study also resulted in the increased production of collagen types I and III in keratinocytes and fibroblasts, and enhanced synthesis of collagen types IV and VII in keratinocytes [26, 27].

Our study is limited by the fact that there was no control arm to compare treatment with no intervention or alternate therapy, the study was performed at a single center rather than multiple centers, and the trial included a small number of patients, particularly for those with superficial skin hydration results. The body mass index and body weight of participants, which may confound changes in soft tissue volume, was also not recorded at each visit. Additionally, despite a study design that included other Asian ethnicities, only participants of Chinese ethnicity enrolled in the study. Future studies could include prospective studies with a control arm and a larger sample size of patients from a broader range of Asian ethnic groups.

## Conclusions

Despite study limitations, our study demonstrated that two treatment sessions of Profhilo^®^ administered four weeks apart for facial skin regeneration in Chinese patients were efficacious, resulting in a decrease in FVLS, WSRS, and the skin elasticity parameter, *R*3 (indicating the skin’s “tiring effect”) over time, and a trend toward a decrease in *F*1 (indicating the degree of skin elasticity) over time, as well as a statistically significant increase in superficial skin hydration and a statistically significant reduction in TEWL at Week 12 versus baseline. Patients in the study rated their tolerability to the product as “good” on a scale from “bad” to “excellent.”

## Data Availability

The author confirms that the data supporting the findings of this study are available within the article. The raw data supporting the conclusions of this article will be made available by the authors upon reasonable request.
